# Heating performance of a laboratory pilot-plant combining heat exchanger and air scrubber for animal houses

**DOI:** 10.1038/s41598-021-86159-5

**Published:** 2021-03-25

**Authors:** Manuel S. Krommweh, Wolfgang Büscher

**Affiliations:** Institute of Agricultural Engineering, University of Bonn, Nußallee 5, 53115 Bonn, Germany

**Keywords:** Renewable energy, Climate change, Climate sciences, Environmental sciences, Climate-change mitigation

## Abstract

Exhaust air treatment systems (EATS) are used in animal husbandry to reduce emissions. However, EATS are associated with high acquisition and operating costs. Therefore, a plant technology is being developed that integrates a recuperative heat exchanger into a biological air scrubber. The overall aim is to reduce total costs of livestock buildings with EATS by saving heating costs and to improve animal environment. In this study, a special pilot-plant on a small-scale, using clean exhaust air, was constructed to evaluate the heating performance on laboratory scale. Three assembly situations of the heat exchanger into trickle-bed reactor were part of a trial with two different defined air flow rates. In all three assembly situations, preheating of cold outside air was observed. The heating performance of the assembly situation with the sprayed heat exchanger arranged below showed an average of 4.4 kW at 1800 m^3^ h^−1^ (outside air temperature range 0.0–7.9 °C). This is up to 18% higher than the other two experimental setups. The heating performance of the pilot-plant is particularly influenced by the outside air temperature. Further research on the pilot-plant is required to test the system under field conditions.

## Introduction

It is common knowledge that intensive animal husbandry, as it is practised in parts of Europe, is associated with certain environmental loads and contributes to air pollution^1,2^. The pollutants emitted from animal husbandry includes particulate matter (PM), ammonia (NH_3_) and odour, and greenhouse gases (GHGs) such as methane (CH_4_) and nitrous oxide (N_2_O)^3–7^. Several studies have shown that the use of exhaust air treatment systems (EATS) on mechanically ventilated livestock buildings for pigs and poultry allows a reduction in PM, NH_3_, and/or odour emissions (end-of-pipe reduction technique). In practise, different plant systems are applied, such as biofilter^8–12^, trickle-bed reactor/air scrubber^13–15^, acid scrubber^14,16^, multiple stage techniques^17–21^ as well as dry filter for separation of PM^22,23^. A widespread overview of the different EATSs (functional principle, design, specification for dimensioning, removal efficiency, general requirements) is given by^5,24–26^.

According to^25^, adequate dimensioning and proper operation of EATSs is very important to ensure effective and continuous emission reduction^24,26^. Even if these conditions are fulfilled, exhaust air treatment is connected with high investment and running costs^27^ and further research is needed to reduce costs^19,28^. For trickle-bed reactors as well as two and three stage treatment systems applied in pig fattening^25^, quotes an annual expenditure of 13–17 € per animal place (ap). Calculations by^29^ mention annual costs for different EATSs applied in pig fattening and sow keeping: 11.1 € ap^−1^ and 26.8 € ap^−1^, respectively, for 1-stage chemical scrubber; 19.1 € ap^−1^ and 55.9 € ap^−1^, respectively, for 3-stage system; 6.6 € ap^−1^ and 16.2 € ap^−1^, respectively, for biofilter.

Regulatory requirements and guidelines on airborne emissions from intensive livestock production are well described by^26,28^. The current version of the “Best Available Techniques (BAT) Reference Document for the Intensive Rearing of Poultry or Pigs” lists EATSs as BAT, but at various points it is noted that “This technique may not be generally applicable due to the high implementation cost”^5^. Nevertheless, in many cases, especially in regions with a high density of livestock, usage of EATSs is essential because farmers are not granted permission to build pig houses without EATS. Furthermore, in Germany, EATSs usually have to be designed such that the total required maximum summer exhaust air flow rate is treated according to DIN 18910^30^. In this context, the high investment and running costs of these systems leads to the requirement of management to reduce the total costs. To operate a livestock building with EATS more efficiently, one research approach is the integration of a heat recovery unit in order to save energy.

Heat recovery technologies are not only an important topic for domestic and industrial buildings to save energy^31–33^, but also to save energy in agricultural buildings. In mechanically ventilated livestock buildings, among others, recuperative air-to-air heat exchangers are applied to transfer the thermal energy from warm exhaust air onto cold air from outside without mixing the two^34,35^. In literature, however, there are hardly any studies on the use of recuperative heat exchangers in livestock buildings. First scientific investigations on the use of a recuperative heat exchanger in pig fattening in East Germany are described by^36^. This is followed by scientific investigations of a recuperative air-to-air heat exchanger by^37,38^. On annual average, heating performance of the heat exchanger investigated in piglet rearing was 18.7 kW ± 8.3 kW and supply air heating 4.9 K ± 2.3 K (at an air flow rate of 11,471 m^3^ h^−1^ ± 3041 m^3^ h^−1^). In addition, there are two test reports of the German Agricultural Society (DLG) on heat exchangers. Under winter conditions, the heating performance of a heat exchanger used in pig fattening is given as 26.0 kW under winter conditions at an air flow rate of 11,465 m^3^ h^−1^^39^. The heating performance of the heat exchanger “Earny” used in broiler fattening is given as 23.8 kW under winter conditions at an air flow rate of 5008 m^3^ h^−1^^40^.

The efficiency of a recuperative air-to-air heat exchanger is indicated by the temperature efficiency^39,41^ (see “Calculation of the temperature efficiency”) and heating performance (see “Calculation of the heating performance”). According to^41^, the temperature efficiency $\Phi_{t}$ “indicates the relationship between the temperature change in the outside air of a heat recovery and the maximum possible temperature change, the difference between the outside air and the exhaust air temperatures”. The temperature difference describes the transfer of sensible thermal energy from exhaust air onto outside air under dry conditions, i.e. without condensation (cf. Eq. (1) in “Calculation of the temperature efficiency”). When the exhaust air temperature falls below the dew point, additional latent energy is transmitted^41,42^. According to^36^, the temperature efficiency depends on the temperature of exhaust and outside air, as well as air mass flow rate: with increasing air mass flow rate, the temperature efficiency decreases rapidly (and system resistance increases)^32^. A further influencing factor is the quality of the heat exchanger: dimension, structure, choice of material, and material thickness (especially in respect to the heat transfer coefficient)^32^. Klement^36^ noted that deposition of dust on the exhaust air side of the heat exchanger surface aggravates not only the heat transfer, but also leads to an increase in pressure resistance with growing dust deposition. To avoid this, the author recommended a water-based cleaning system.

In literature, Zwoll^43^ reports on a research approach on a multiple stage EATS with an integrated heat exchanger unit for broilers^44^. The first treatment stage is composed of vertically mounted tube bundle straighteners sprayed with fluid (pH 3.5) for reduction of PM and NH_3_; the second consists of structured packing material sprayed with scrubbing water (pH 6.8) for reducing odour. Because the PM and NH_3_ emissions during the first few days of poultry fattening are very low (low weight of broilers; low air flow rate), only the second treatment stage is required for cleaning exhaust air. However, at that time, the heat requirement of broilers is very high (the initial indoor temperature is approximately 32–34 °C), which also leads to a very high exhaust air temperature. Therefore, the first stage is used for heat recovery: when warm exhaust air passes through the tube bundle straighteners (not sprinkled at that time) horizontally, outside air is led vertically through it and becomes heated. As a consequence, energy costs can be reduced. This stage of the EATS is used either as a heat recovery system (during the first few days of poultry fattening) or later in the poultry fattening schedule as a treatment stage for cleaning exhaust air, but not both simultaneously.

The long-term objective of this research is the development of a new plant technology for mechanically ventilated livestock buildings which is able to clean exhaust air and recovery heat simultaneously: the *exchange scrubber*. The idea of the exchange scrubber is patented (Patent No. EP1815902, 20.09.2007). The name is a derivative of the terms “heat exchanger” and “air scrubber”. It combines a biological trickle-bed reactor with a recuperative air-to-air heat exchanger in one processing stage to create and use possible synergistic effects: (1) recovery of thermal energy of warm exhaust air by conduction onto cold outside air, which leads to savings of heating energy and therefore conservation of fossil energy sources and reduction of carbon dioxide emissions, (2) purification of exhaust air from PM, NH_3_ and odour (protection of environment and local residence), and (3) reduction of total costs for livestock buildings with exhaust air treatment by substitution of fuel costs.

**Figure 1:**
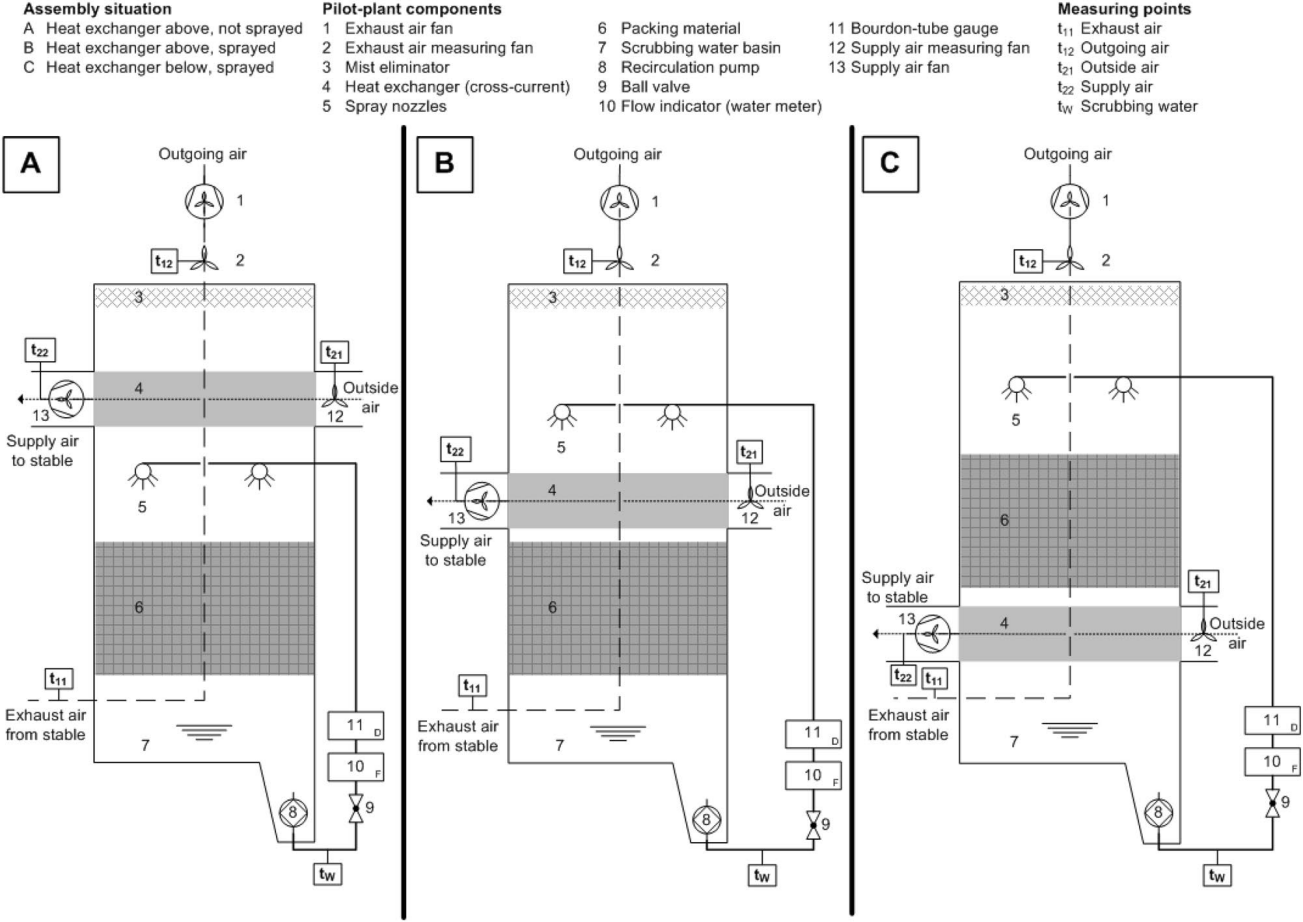
Schematic sketch of the exchange scrubber with the three tested assembly situations of the heat exchanger unit.

Research into the first effect will be shown in this paper. To this end, a special small-scale exchange scrubber pilot-plant on a laboratory scale was constructed. Technology of exhaust air treatment is based on a certified system^45^. The objectives of this partial study were (i) to determine the heating performance of this new plant technology, as well as the influence of outside air and scrubbing water temperature on the heat recovery process and (ii) to examine whether assembly situation—of heat exchanger unit into the trickle-bed reactor (shown in Fig. 1)—affects the level of heat recovery (see details in “Experimental setup and procedure”).

## Results and discussion

### Temperature and heating performance profiles of a single measurement

**Figure 2:**
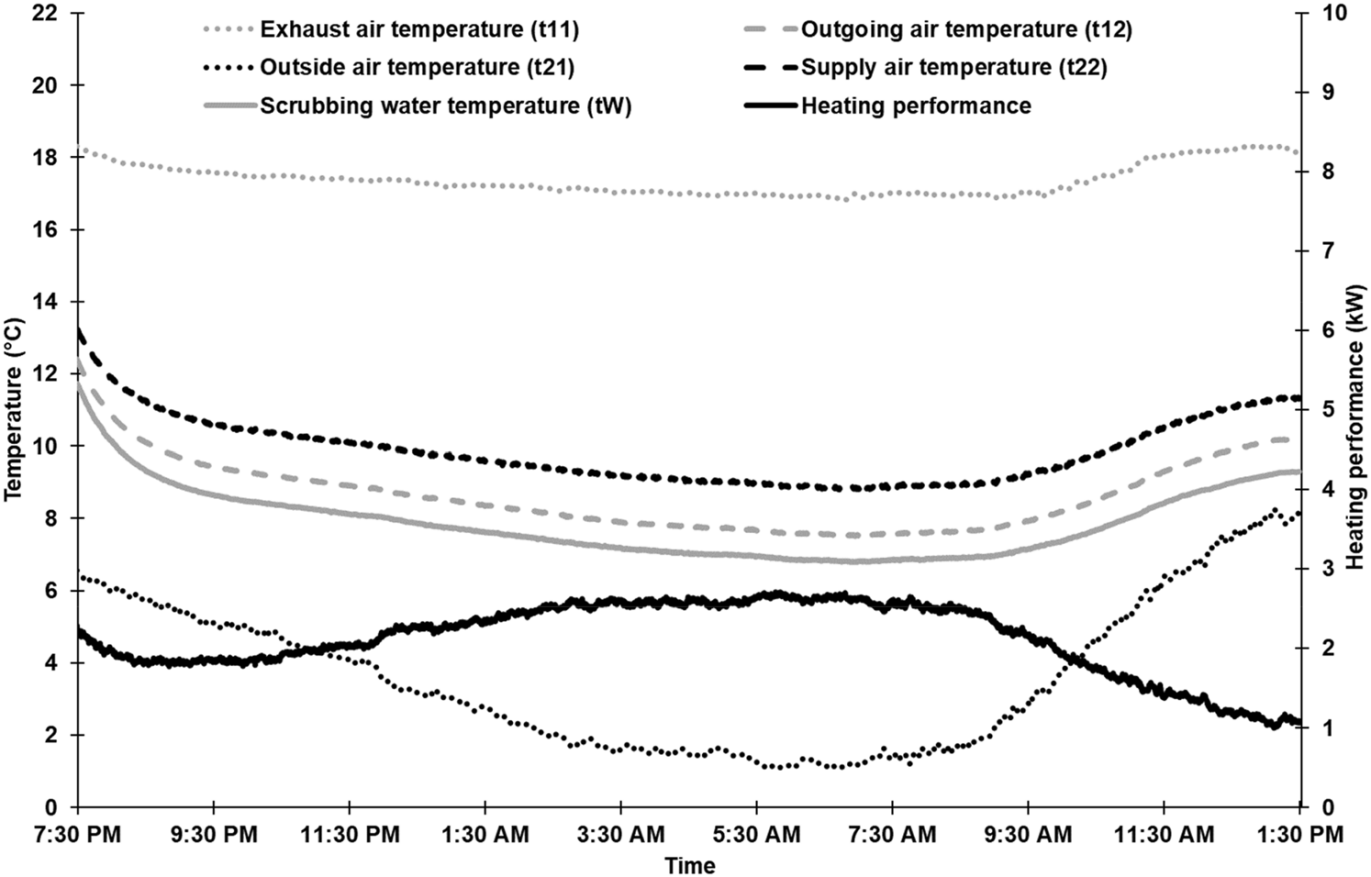
Heating performance and temperature profiles at the measuring points of the exchange scrubber pilot-plant of a single measurement during the experimental period (February 29th/March 1st, 2016; assembly situation B of the heat exchanger at 1000 m^3^ h^−1^).

The temperature profile at the measuring points (cf. Fig. 1) and the heating performance $\dot{Q}$ is shown in Fig. 2 for a single measurement (heat exchanger assembly situation B; 1000 m^3^ h^−1^). During the first hour after starting the pilot-plant, temperatures of scrubbing water, outgoing and supply air decreased rapidly until a certain equilibrium state was reached (adaptation period). This was observed for all measurements. This effect was due to shutting down the pilot-plant between single measurements. Because EATSs operate continuously in practise, the data of the adaptation period were not used in the data analysis.

In Fig. 2, exhaust air entered the pilot-plant with an average temperature of 17.3 °C (relative humidity, RH, 30.2%) and left it with a temperature of 8.4 °C (RH 99.6%). Thus, exhaust air was cooled down by 8.9 K while passing through the pilot-plant. The high RH of outgoing air was due to the trickled water inside the plant and has also been observed in conventional EATSs by^20,45^. Outgoing air temperature was similar to the scrubbing water temperature, with a mean temperature of 7.6 °C, which is in agreement with previous results^20^. The average outside air and supply air temperatures were 3.3 °C (RH 61.7%) and 9.7 °C (RH 43.2%). On passage through the recuperative heat exchanger unit, outside air was warmed up by an average of 6.4 K. RH of incoming air decreased because no moisture transfer by the heat exchanger unit was possible (see“Description of the small-scale exchange scrubber pilot-plant”). The maximum preheating effect, shown in Fig. 2, was 7.9 K at an outside air temperature of 1.1 °C early in the morning. In practise, for a single recuperative heat-exchanger, Rösmann^35,37^ reported a similar preheating effect and a similar temperature difference between outside air and exhaust air, but at obviously higher air flow rates. Furthermore, Rösmann^37^ ascertained that the thermal output of the exhaust air side was always higher than the thermal absorption of the supply air side at equal exhaust and supply air flow rates, based on temperature data. In this study, this was also observed on the exchange scrubber pilot-plant. The heating performance and temperature efficiency (Fig. 2) were 2.2 kW and 0.47 on average.

### Influence of outside air and scrubbing water temperature on heating performance

**Figure 3:**
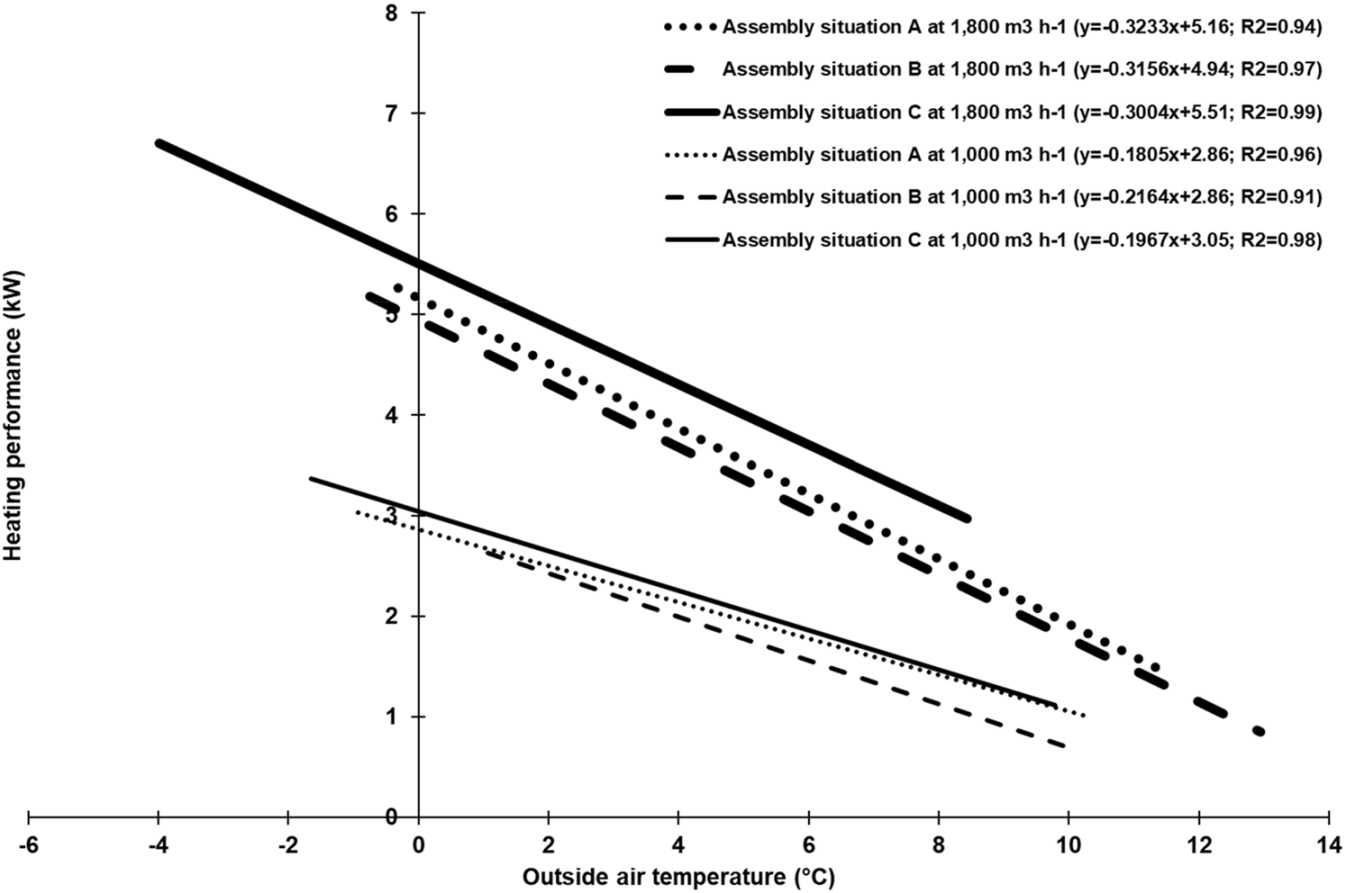
Relationship between heating performance of the exchange scrubber pilot-plant and outside air temperature at different assembly situations of the heat exchanger and different air flow rates (n = 20,400 per each experimental setup).

As Fig. 2 indicates, heating performance inversely depended on outside air temperature: if outside air temperature decreased, heating performance increased and vice versa. This relationship is shown in Fig. 3 for all six experimental setups. For clarity, the regression lines, but not the single data points, are shown. This figure also shows a strong negative correlation: the lower the outside air temperature, the higher the heating performance of the exchange scrubber and vice versa. This relationship is in agreement with previous results on a single recuperative heat-exchanger in a piglet-rearing barn^35,36^. In addition, our own research on another outside air preheating system—a modular housing system for fattening pigs with an integrated geothermal heat exchanger—showed a similar relationship between heating performance and outside air temperature^46^.

**Figure 4:**
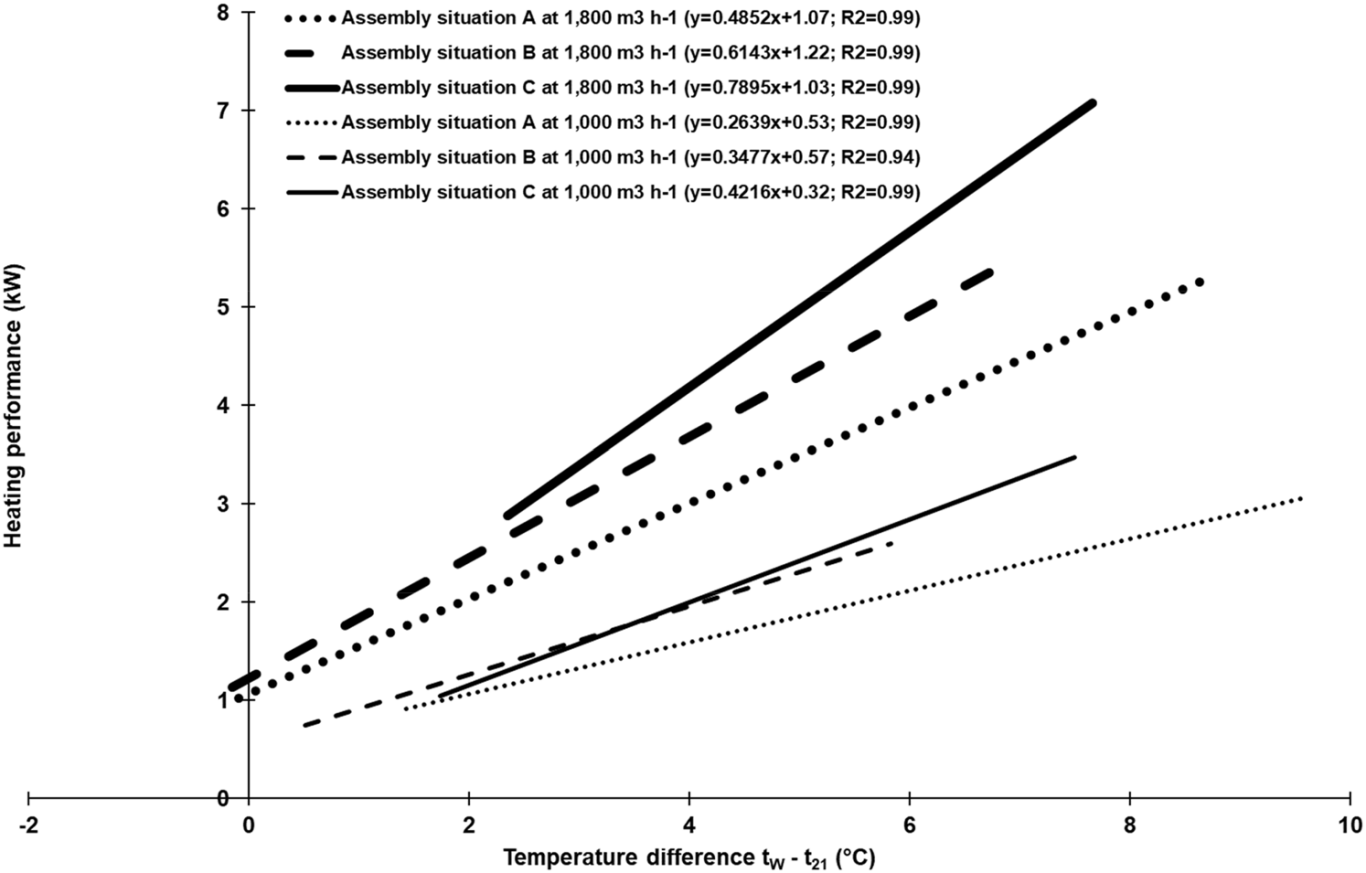
Relationship between heating performance of the exchanger scrubber pilot-plant and the temperature difference between scrubbing water and outside air (Δ t_W_–t_21_) at different assembly situation of the heat exchanger and different air flow rates (n = 20,400 per each experimental setup).

Figure 2 shows that the temperature profiles of scrubbing water and supply air run in parallel to one another: if outside air temperature decreased at night, temperatures of scrubbing water and supply air decreased likewise. Before noon, the opposite was true. An explanation of this pattern is that at decreasing temperatures of incoming outside air, the temperature difference between exhaust air, as well as the scrubbing water and outside air temperatures, increases. As a consequence, temperatures of exhaust air and scrubbing water decrease because thermal energy is transferred convectively to the incoming outside air. This suggests that the heating performance of the exchange scrubber might depend on the outside air temperature, but also indirectly on the scrubbing water temperature. To verify this, the difference between the temperature of scrubbing water and outside air temperature was calculated (Δ t_W_–t_21_) for each data set. Figure 4 shows the heating performance of the exchange scrubber for all six experimental setups as a function of Δ t_W_–t_21_. Again, regression lines but no single data points are shown. However, a strong positive correlation is clearly seen for all experimental setups, with high coefficients of determination. Thus, the abovementioned assumption is confirmed: the greater the Δ t_W_–t_21_, the higher the heating performance of the exchange scrubber and vice versa. This means that if the outside air temperature exceeds the scrubbing water temperature (in this case, Δ t_W_–t_21_ has a negative value), cooling effects might be expected (cf. Fig. 4).

### Temperature efficiency

**Figure 5:**
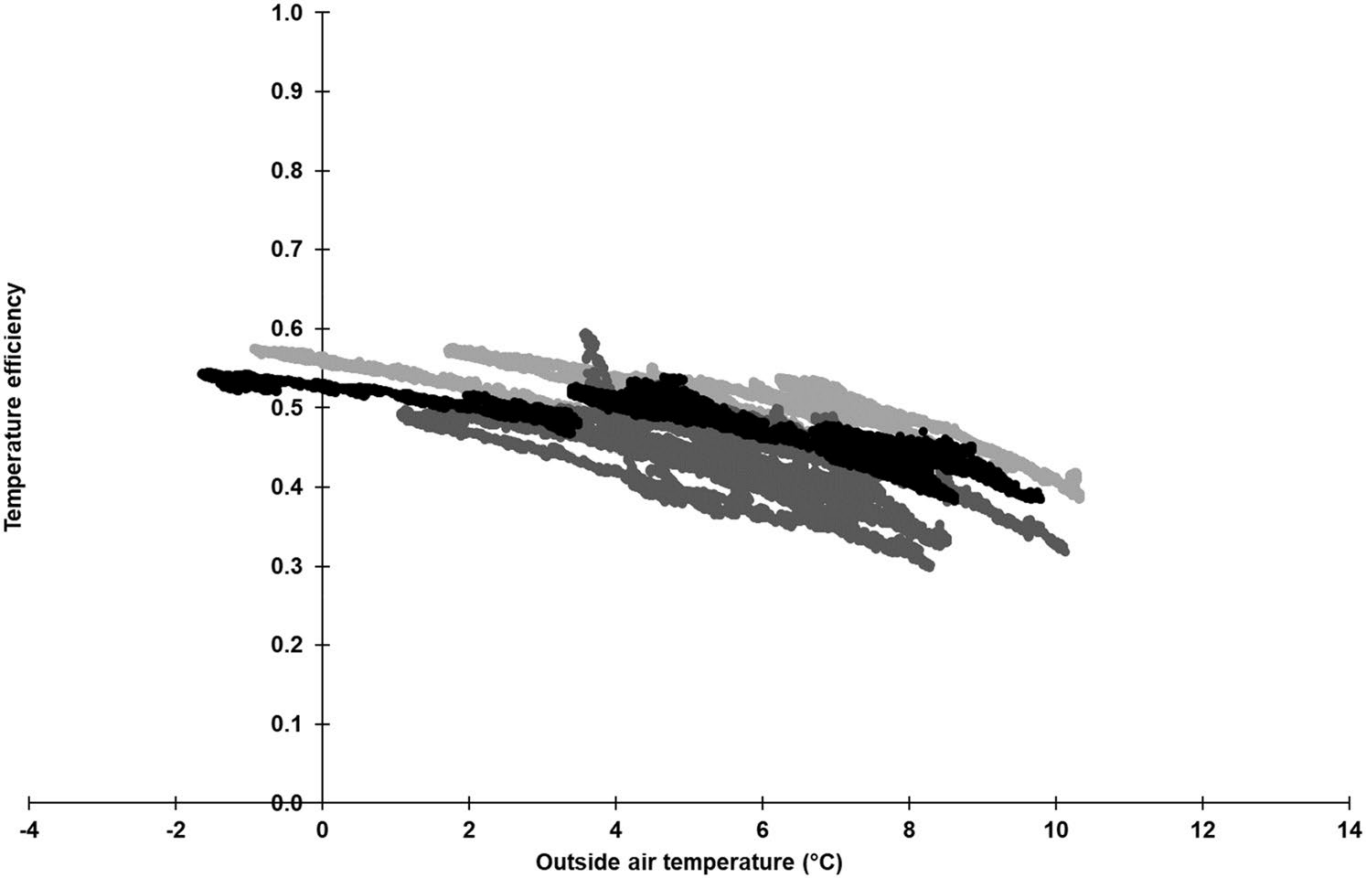
Temperature efficiency of the heat exchanger scrubber pilot-plant dependent on outside air temperature at an air flow rate of 1000 m^3^ h^−1^. Light grey dots: assembly situation A; dark grey dots: assembly situation B; black dots: assembly situation C; n = 20,400 per each experimental setup.

Depending on the outside air temperature, the temperature efficiency, Φ_t_, of the pilot-plant is shown in Fig. 5 for the experimental setups at 1000 m^3^ h^−1^. The values range between 0.30 and 0.58 depending on the assembly situation of heat exchanger unit. This shows that assembly situation A had the highest value for Φ_t_. This could be due the heat exchanger unit not being sprayed (see “Most effective assembly situation of heat exchanger unit”). However, all three assembly situations demonstrated a similar profile: the higher the outside air temperature, the stronger the decrease in Φ_t_, but this relationship does not seem to be linear. Previous research, on a conventional recuperative heat exchanger by^39^, showed a similar nonlinear relationship in this range of outside air temperature, whereby Φ_t_ decreased rapidly at an outside air temperature of 7 °C, but, at around 12 °C, there was no heating performance. The authors stated a mean Φ_t_ of 0.42 and that the outside air was heated by 6.8 K on average. At the heat exchanger “Earny” used in broiler fattening^40^, Φ_t_ was 0.57 and outside air was heated by 12.6 K. Klement^36^ reported a heat exchanger installed in a pig fattening house with Φ_t_ between 0.20 and 0.30.

In general, the temperature efficiency should be treated with caution in respect to a direct system comparison. The operating states of the various investigated heat exchangers, for example, plant size, supplied air flow rate, as well as the temperature difference between exhaust and outside air, are important influencing factors that are often different, especially in practical scale. In addition, in this study, the temperature efficiency did not consider the influence of scrubbing water within the exchange scrubber. Nevertheless, Φ_t_ allows a first assessment of the examined system.

### Most effective assembly situation of heat exchanger unit

Because the outside air temperature ranges of the single experimental setups are different (cf. Fig. 3), the performance comparison of the three assembly situations of the heat exchanger unit (Fig. 1) is based on outside air temperature ranges where data are available for all three experimental setups: 2.0–9.9 °C and 0.0–7.9 °C for measurements with constant air flow rates of 1000 m^3^ h^−1^ and 1800 m^3^ h^−1^.

In Table 1, the core values for defined outside air temperature ranges are shown for each experimental setup. As expected, it can be seen in each column that, with a decrease in outside air temperature, outside air preheating, temperature efficiency and heating performance increased and scrubbing water temperature decreased (cf. Fig. 3). The differences of heating performance between the three assembly situations are more distinct for the higher air flow rate. At an air flow rate of 1000 m^3^ h^−1^, the heating performance of C (1.9 kW) was 5% higher than assembly situation A (1.8 kW) and 16% higher than B (1.6 kW). At the higher air flow rate of 1800 m^3^ h^−1^, the heating performance of assembly situation C (4.4 kW) was 11% higher than assembly situation A (3.9 kW) and 18% higher than B (3.6 kW). Therefore, on the basis of the considered outside air temperature ranges, assembly situation C of the heat exchanger unit was the most effective experimental setup with respect to outside air preheating and heating performance, followed by A and B. This could be explained by the fact, that assembly situation C has warm exhaust air entering the exchange scrubber, which is led directly through the heat exchanger unit. In contrast, assembly situation B has warm exhaust air leading through the packing material before going through the heat exchanger unit (cf. Fig. 1). In this case, warm exhaust air needs more time to reach the heat exchanger and, meanwhile, is cooled down more by scrubbing water. Here, the transfer of thermal energy might arise primarily from scrubbing water. In assembly situation C, transfer of thermal energy could arise from both scrubbing water and exhaust air. In assembly situation A, thermal energy is not removed from scrubbing water, but only from water-saturated exhaust air because the heat exchanger unit is not sprayed. This is the reason for the scrubbing water temperature on assembly situation A being an average of up to 2 K higher in comparison to the assembly situations B and C (Table 1).

**Table 1:** Performance comparison of the exchange scrubber pilot-plant for the three assembly situations of heat exchanger unit and air flow rates of 1000 m^3^ h^−1^ and 1800 m^3^ h^−1^ by stating the core values (mean ± standard deviation): scrubbing water temperature, t_W_; outside air preheating, Δ t_22_–t_21_; temperature efficiency, Φ_t_; heating performance,$\dot{Q}$; ns = not specified.

Outside air temperature (t_21_) range (°C)	Air flow rate: 1000 m^3^ h^−1^			Air flow rate: 1800 m^3^ h^−1^		
	Assembly situation heat exchanger unit			Assembly situation heat exchanger unit		
	A	B	C	A	B	C
**Total**^a^						
t_W_ (°C)	10.8	8.8	9.6	9.8	7.8	8.1
Δ t_22_–t_21_ (K)	5.7	4.7	5.8	6.4	5.8	6.8
Φ_t_	0.50	0.42	0.47	0.51	0.45	0.49
$\dot{Q}$ (kW)	1.8	1.6	1.9	3.9	3.6	4.4
**0.0–1.9**						
t_W_ (°C)	ns	ns	ns	8.7 ± 0.3	7.0 ± 0.3	6.2 ± 0.5
Δ t_22_–t_21_ (K)	ns	ns	ns	8.0 ± 0.3	7.4 ± 0.3	8.0 ± 0.2
Φ_t_	ns	ns	ns	0.53 ± 0.01	0.50 ± 0.01	0.50 ± 0.02
$\dot{Q}$ (kW)	Ns	ns	ns	4.8 ± 0.2	4.7 ± 0.2	5.2 ± 0.1
**2.0–3.9**						
t_W_ (°C)	9.9 ± 0.5	7.7 ± 0.3	8.0 ± 0.5	9.2 ± 0.5	7.5 ± 0.3	7.4 ± 0.4
Δ t_22_–t_21_ (K)	7.7 ± 0.5	6.4 ± 0.4	7.2 ± 0.3	6.8 ± 0.3	6.3 ± 0.4	7.3 ± 0.3
Φ_t_	0.55 ± 0.02	0.47 ± 0.01	0.50 ± 0.01	0.52 ± 0.02	0.48 ± 0.01	0.50 ± 0.01
$\dot{Q}$ (kW)	2.4 ± 0.2	2.2 ± 0.2	2.4 ± 0.1	4.1 ± 0.2	3.9 ± 0.3	4.7 ± 0.2
**4.0–5.9**						
t_W_ (°C)	10.4 ± 0.2	8.6 ± 0.4	9.2 ± 0.2	10.4 ± 0.5	8.1 ± 0.4	8.6 ± 0.3
Δ t_22_–t_21_ (K)	6.3 ± 0.3	5.3 ± 0.4	6.4 ± 0.4	6.2 ± 0.5	5.3 ± 0.5	6.5 ± 0.3
Φ_t_	0.52 ± 0.01	0.45 ± 0.02	0.50 ± 0.01	0.52 ± 0.02	0.45 ± 0.02	0.48 ± 0.01
$\dot{Q}$ (kW)	2.0 ± 0.1	1.8 ± 0.2	2.1 ± 0.1	3.8 ± 0.3	3.3 ± 0.3	4.1 ± 0.2
**6.0–7.9**						
t_W_ (°C)	11.1 ± 0.3	9.2 ± 0.4	10.3 ± 0.3	10.7 ± 0.2	8.5 ± 0.2	10.1 ± 0.3
Δ t_22_–t_21_ (K)	5.2 ± 0.4	4.3 ± 0.4	5.1 ± 0.3	4.7 ± 0.5	4.0 ± 0.4	5.4 ± 0.3
Φ_t_	0.50 ± 0.03	0.41 ± 0.03	0.45 ± 0.02	0.48 ± 0.02	0.39 ± 0.02	0.47 ± 0.01
$\dot{Q}$ (kW)	1.7 ± 0.1	1.4 ± 0.1	1.6 ± 0.1	2.9 ± 0.3	2.5 ± 0.2	3.4 ± 0.2
**8.0–9.9**						
t_W_ (°C)	11.5 ± 0.2	9.8 ± 0.4	11.0 ± 0.3	ns	ns	ns
Δ t_22_–t_21_ (K)	3.8 ± 0.4	3.1 ± 0.3	4.4 ± 0.3	ns	ns	ns
Φ_t_	0.44 ± 0.02	0.36 ± 0.03	0.43 ± 0.02	ns	ns	ns
$\dot{Q}$ (kW)	1.2 ± 0.1	1.0 ± 0.1	1.4 ± 0.1	ns	ns	ns

^a^Weighted average. For variants with air flow rate of 1000 m^3^ h^−1^, data sets with outside air temperatures from 2.0 to 9.9 °C were considered. For variants with air flow rate of 1800 m^3^ h^−1^, data sets with outside air temperatures from 0.0 to 7.9 °C were considered.

Even if assembly situation C was the most effective assembly situation in this study, where clean exhaust air was used, it is questionable whether it will be also the most effective in practical scale. Exhaust air from livestock buildings contain, among other things, dust that shall be separated by the trickle-bed reactor. It would be conceivable that dust would deposit and microorganisms would settle on the sheets of the heat exchanger, which could lead to a reduction in thermal energy transfer^36^.

In general, in considering the dimensions of the heat exchanger unit and the mild winter conditions at the trial site, the heating performance of the exchange scrubber is positively assessed. In colder outside air temperatures (t_21_ < 0 °C) and the same air flow rate, higher outside air preheating, along with higher heating performances are expected (cf. Eq. (2); Fig. 3^37^).

A direct system comparison of this exchange scrubber pilot-plant on a laboratory scale to already published data of heat exchanger investigated in practical scale by^36–40^ is not reasonable, because the operating states of the various investigated heat exchangers, for example, plant size, heat exchanger volume, supplied air flow rate, as well as the temperature difference between exhaust and outside air, are important influencing factors that are often different, especially in practical scale. For a direct system comparison, it is necessary to examine all systems to be compared on a test bench under exactly the same input variables as described in^41,47^.

### Advantages and disadvantages of the exchange scrubber technique

By integrating the heat exchanger into the trickle-bed reactor, the exchange scrubber is able to transfer heat energy from the exhaust air and the circulating scrubbing water to the cold incoming outside air. From an animal welfare point of view, this effect of supply air heating is particularly positive on winter days with very low outside air temperatures^37,39,46^. Cold, incoming air can, under certain circumstances, lead to cold symptoms in the animals because it falls directly into the animal area due to its density. This effect is significantly reduced by preheating of incoming air. Another advantage of the exchange scrubber is that heat energy that would otherwise have been discharged unused into the atmosphere can now be recovered proportionally through the heat exchanger and used again to heat the barn. As a result, heating energy savings are expected, which are not only financially beneficial for the farmer, but also conserve other energy sources (usually fossil fuels). This also avoids CO_2_ emissions.

This is offset by the fact that the EATS will be somewhat costlier to produce due to the integration of the heat exchanger. The heat exchanger, like the EATS, must be planned and dimensioned on a case-by-case basis according to the requirements of the corresponding barn building. In future, practical tests must in any case consider the extent to which possible contamination of the heat exchanger will affect heat recovery. This also requires recording the power consumption required to operate the exchange scrubber.

## Conclusions

This study on the exchange scrubber pilot-plant leads to the following conclusions:

1. The new exchange scrubber technology is able to preheat cold incoming outside air. On practical scale, this could lead to a reduction in heating costs, use of fossil fuels and thereby carbon dioxide emissions.
2. On laboratory scale with clean exhaust air, assembly situation C with the sprayed heat exchanger below the packing material generated the highest heating performance. Further studies are required to examine whether this is also the case in field conditions.
3. As with conventional recuperative heat exchangers, the heating performance of the exchange scrubber depends primarily on the outside air and, indirectly, on scrubbing water temperature.
4. The preheating effect of incoming outside air might have a positive influence on the barn climate.

## Methods

### Description of the small-scale exchange scrubber pilot-plant

The internal floor area of the small-scale exchange scrubber pilot-plant was 1.44 m^2^ (1.2 m × 1.2 m) and the total height was 5.55 m. The framework consisted of four crossing piers (stainless steel double-U-profiles). The sidewalls were constructed of polyvinyl chloride (PVC) hollow chamber panels (panel height: 200 mm, panel thickness: 30 mm; Alfons Greten Betonwerk GmbH & Co. KG, Essen/Oldenburg, Germany). The panels were tongued and grooved so that the sidewalls were hermitically sealed against ambient air. On all four sides of the pilot-plant, the lower two rows of hollow chamber panels were perforated for allowing exhaust air to enter the plant (total exhaust air entry surface: 0.7 m^2^). The sidewall structure was made by guiding each single panel from the top in the U-profile. In this way, a fast assembly and disassembly respective to modifications of the pilot-plant was possible (cf. Fig. 1). On the top, the pilot-plant was closed by a wooden board (thickness 10 mm) with a centrally arranged hole for the vent stack (inner diameter: 520 mm, height: 600 mm). The exhaust air fan and the measuring fan were installed in this vent stack. From the top of the vent stack the exhaust air was led by a flexible tube to the outside. Outside air was also guided by another flexible tube from outside through an air duct (inner diameter: 520 mm) into the heat exchanger unit. The warmed air left it by passing a second air duct where the supply air fan was located (Fig. 1). Exhaust and supply air fans were axial fans with a diameter of 500 mm (both type FC050-4DQ.4F.A7; Ziehl-Abegg, Künzelsau, Germany). For each fan a frequency converter was used (type ER22-3.0/4G; BLEMO Frequenzumrichter, Rodgau, Germany).

The entire exchange scrubber pilot-plant was stationary in a water basin (1.75 m × 1.75 m; total height: 0.85 m; basin deepness: 0.6 m) with a centrally arranged plug hole. By the basin, the circulating scrubbing water was intercepted and guided by the plug hole into a sump pit. There, a recirculation pump (GH-DP 6315 N, Einhell Germany AG, Landau/Isar, Germany) was positioned, which conveyed the scrubbing water continuously through a hose system to the liquid distributors (spray nozzles) into the plant. The hose system was equipped with a ball valve, water meter, and bourdon-tube gauge (indicating range: 0–4 bar; measuring accuracy: ± 1.6%). To ensure a uniform scrubbing water distribution, there were four spray nozzles (tangential-flow full cone nozzle; polyvinylidine fluoride (PVDF); beam angle: 120°; type 422.888 5E CE; Lechler GmbH, Metzingen, Germany) installed with a distance of 0.32 m to the packing material respective to the heat exchanger, dependent on the experimental setup (Fig. 1). The trickling density was continuously adjusted to 1.11 m^3^ [scrubbing water] m^−2^ [filter area] h^−1^. The entire amount of scrubbing water in the pilot-plant was 400 L.

Inside the pilot-plant, there were four layers of packing material made of polypropylene (PP). Following the test report of the aforementioned biological trickle-bed reactor^45^, the bottommost layer (0.225 m height; grid structure) had a specific packing material surface area of 150 m^2^ m^−3^, the second layer (0.3 m height; cross-fluted structure) had an area of 120 m^2^ m^-3^ and the third and fourth layers (each 0.3 m height; cross-fluted structure) both had 240 m^2^ m^−3^ (RIMU Agrartechnologie GmbH, Königsbrunn, Germany). The mist eliminator with a height of 0.125 m was also made of PP (2H PP-Drift Eliminator TEP 130; GEA 2H Water Technologies GmbH, Wettringen, Germany).

The applied plate heat exchanger unit (1200 mm × 1200 mm × 600 mm) was made of crossways waveform structured double folded sheets. These sheets are made of PVC by a thermoforming process (deep-drawing). The sheet thickness was 0.6 mm, the distance between the single sheets (in total 53) on the inlet and outlet area was 22 mm. The exchange surface was 102.96 m^2^ in total (≙ 119.17 m^2^ m^−3^). This heat exchanger was a direct recuperative cross-flow model: exhaust air was led vertically through the heat exchanger, while outside air was led through horizontally. The heat transfer that ensued was convective over the PVC sheets exclusively. There was no mixing of exhaust and outside air and therefore no moisture transfer. Since this study was carried out in the laboratory for machines of the Institute of Agricultural Engineering, only clean air was available. As a result, no measurement could be done for removal efficiency (e.g. ammonia).

The exchange scrubber pilot-plant was a module of a full-scale facility and can be expanded optionally, as required according to^30^ (cf. “Introduction”). The used exhaust air cleaning technique has been certified for a maximum filter surface load of 2800 m^3^ [exhaust air] m^−2^ [filter surface area] h^−1^^45^. Considering the filter surface area of the exchange scrubber pilot-plant stated above, a maximum air flow rate of 4032 m^3^ h^−1^ can be achieved. Thus, the dimensioning of the pilot-plant was adequate for 81 piglets with a live weight of 30 kg (49.4 m^3^ piglet^−1^ h^−1^ according^30^). Therefore, the dimensions of the heat exchanger unit were made according to this.

### Experimental setup and procedure

Three assembly situations of the heat exchanger unit into the trickle-bed reactor arose from the abovementioned second objective (A, B, and C; Fig. 1). These were investigated in each case at two different defined air flow rates of 1000 m^3^ h^−1^ and 1800 m^3^ h^−1^. This resulted in six experimental setups. The exhaust and supply air fans were both adjusted to deliver the same amount of air in parallel. The pilot-plant was run in cold outside temperatures during winter. Each measurement lasted for 18 h and each test series comprised five repeated measurements. The experiments were carried out at the laboratory for machines at the Institute of Agricultural Engineering (University of Bonn, Germany) in the winter periods of 2014/2015 and 2015/2016.

### Detection of temperature and humidity

The temperatures of exhaust (t_11_), outgoing (t_12_), outside (t_21_), and supply (t_22_) air, as well as scrubbing water (t_W_), were measured at the corresponding measuring points, as shown in Fig. 1, and recorded by a data logger at intervals of 15 s. For exhaust, outgoing, outside, and supply air, combined temperature and humidity sensors were applied (sensor type FHA646E1C; measuring range was − 20 to + 80 °C for temperature and 0–100% for relative humidity (RH) of the air; Ahlborn Mess- und Regeltechnik GmbH, Holzkirchen, Germany). These sensors used negative temperature coefficient thermistors, with an accuracy of ± 0.4 °C in the temperature range − 20 to 0 °C, and ± 0.1 °C in the temperature range 0–70 °C. The capacitive humidity sensor had an accuracy of ± 2% RH. Scrubbing water temperature was measured by Pt100 sensors (resistor-based sensors; measuring range of − 200 to + 400 °C; measuring accuracy of ± 0.3 °C; Ahlborn Mess- und Regeltechnik GmbH, Holzkirchen, Germany).

### Measurement and calculation of air flow rate

The air flow rate $(\dot{V})$ of supply and exhaust air was determined by calibrated measuring fans (the diameter in each case was 520 mm; measuring ranges, as specified by the manufacturer, are 450–9000 m^3^ h^−1^; Reventa GmbH & Co. KG, Horstmar, Germany), which has been shown to be the most accurate measurement method^48^. Both measuring fans were installed upstream with respect to the fan (Fig. 1). This position allows the highest measurement accuracy^49,50^. A data logger recorded the air flow rate in cubic meter air per hour (m^3^ h^−1^) of each measuring fan at intervals of 15 s.

### Calculation of the temperature efficiency

The temperature efficiency was calculated using Eq. (1)^41,47^.

1 $$\Phi_{t} = \frac{{t_{22} - t_{21} }}{{t_{11} - t_{21} }}$$

where, $\Phi_{t}$ = temperature efficiency, $t_{11}$ = exhaust air temperature (raw gas) (°C), $t_{12}$ = outgoing air temperature (clean gas) (°C), $t_{21}$ = outside air temperature (°C), $t_{22}$ = supply air temperature (°C).

### Calculation of the heating performance

The heating performance $\dot{Q}$ of the exchange scrubber was calculated using the following equation based on^47^:

2 $$\dot{Q}\; ({\text{kW}}) = \frac{{\dot{m} \; c_{pl} \;(t_{22} - t_{21} )}}{1000}$$

where, $\dot{Q}$ = heating performance (kW), $\dot{m}$ = air mass flow rate (kg h^−1^), $c_{pl}$ = specific heat capacity of air with regards to dry air (Wh kg^−1^ K^−1^) ($c_{pl}$ = 1.005 kJ kg^−1^ K^−1^ = 0.28 Wh kg^−1^ K^−1^), $t_{21}$ outside air temperature (°C), $t_{22}$ supply air temperature (°C).

The air mass flow rate $\dot{m}$ was computed using Eq. (3) by air flow rate $\dot{V}$ and density of air $\rho_{air}$.

3 $$\dot{m} \;({\text{kg }}\;{\text{h}}^{ - 1} ) = \dot{V} \;\rho_{air}$$

### Measurement data analysis

The data analysis and result presentation were done using Microsoft Office Professional 2013 and Microsoft Office Professional Plus 2019 as well as IBM SPSS Statistics 24. Figure 1 was created with Microsoft Office Visio 2007.

## Data Availability

The datasets used and/or analysed during the current study are available from the corresponding author on reasonable request.
